# Current advances in using neurotrophic factors to treat neurodegenerative disorders

**DOI:** 10.1186/2047-9158-1-14

**Published:** 2012-07-26

**Authors:** April M Weissmiller, Chengbiao Wu

**Affiliations:** 1 Department of Neurosciences, University of California San Diego, School of Medicine, La Jolla, CA, 92093-0649, USA; 2 Department of Neurosciences, University of California San Diego, George Palade Labs (GPL), Room 337 MC-0649, 9500 Gilman Drive, La Jolla, CA, 92093, USA

**Keywords:** Neurotrophin, Neurotrophic factor, BDNF, NGF, Gene delivery, Mimetics

## Abstract

Neurotrophic factors are best known for their roles in both development and continued maintenance of the nervous system. Their strong potential to elicit pro-survival and pro-functional responses in neurons of the peripheral and central nervous system make them good drug candidates for treatment of a multitude of neurodegenerative disorders. However, significant obstacles remain and need to be overcome before translating the potential of neurotrophins into the therapeutic arena. This article addresses current efforts and advances in resolving these challenges and provides an overview of roadmaps for future translational research and neurotrophin-based drug developments.

## Introduction

Neurotrophic factors or neurotrophins are a group of growth factors which have been classically described for their ability to regulate differentiation and to support growth during development of the vertebrate nervous system. The family of neurotrophins consists of nerve growth factor (NGF), brain-derived neurotrophic factor (BDNF), neurotrophin 3 (NT3), and neurotrophin 4 (NT4). In order to elicit a survival response, each binds to one member of the tyrosine receptor kinase (Trk) family: NGF binds to TrkA, BDNF and NT4 bind to TrkB, and NT3 binds to TrkC. Each of the neurotrophins can similarly respond through an apoptotic pathway initiated by binding to the 75 kD neurotrophin receptor (p75^NTR^). The spatial and temporal balance achieved between neuronal survival and death depends on the overall level of neurotrophin present and the type of receptors that are expressed [1]. In the peripheral nervous system, NGF is the dominant neurotrophic factor, acting on sympathetic and sensory neurons. However, in the central nervous system, BDNF is the predominant neurotrophin utilized due to the abundant expression of TrkB, with NGF providing trophic support specifically to the basal forebrain cholinergic neurons (BFCNs) which express TrkA. Studies from heterozygous mice expressing reduced levels of NGF and BDNF reveal that these two factors are essential for multiple functions throughout adulthood such as proper memory acquisition, memory retention, long-term potentiation, and cholinergic innervation [2,3].

Since the discovery of NGF in the 1950s [4] a large body of experimental data has pointed to multiple roles for the neurotrophins. Firstly, most neurotrophins are required during development and differentiation, during a time which specific synaptic connections are being made and proper circuits are being formed. Secondly, neurotrophin signaling plays an important role in adulthood at a time in which continued maintenance and modulation of those connections is required for normal brain function.

## Neurotrophic signaling pathways

**Figure 1:**
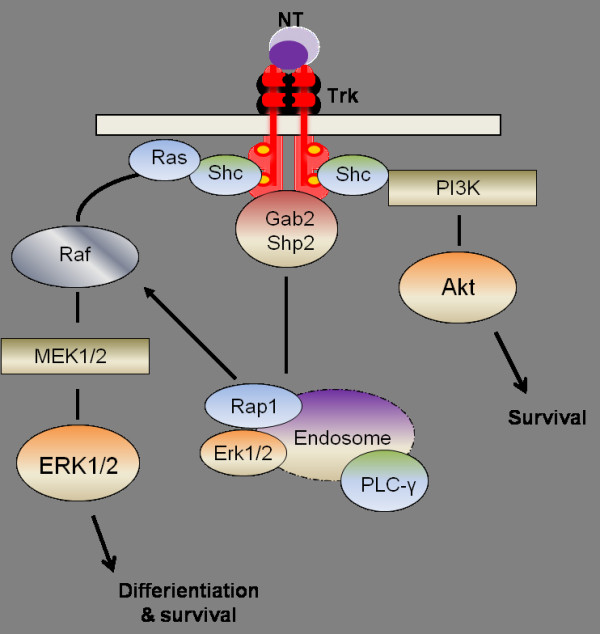
**Trk receptor activation by neurotrophic factors.** Binding of neurotrophic factor causes dimerization and autophosphorylation of the receptor leading to activation of signaling cascades through Src and Shc adaptor proteins which bind the Trk. Once activated Shc increases the activation of Ras, which leads to MAPK/ERK1/2, causing differentiation and survival through transcriptional events, an event that can also occur in signaling endosomes. Shc activation also leads to increases in Akt through activation of PI3K promoting cell survival by inhibiting apoptotic signaling. NT: neurotrophin; Trk: tyrosine kinase receptor; ERK1/2: extracellular signal-regulated kinase; MEK: mitogen-activated protein kinase; PLC-γ: phospholipase Cγ; rap1: ras-related protein1; Shp2: tyrosine phosphatases.

Although different neurotrophins act on different receptors in the brain, both NGF and BDNF elicit pro-survival and pro-functional responses using essentially the same canonical signaling pathways: the mitogen-activated protein kinase (MAPK) pathway, the phosphatidylinositol 3-kinases (PI3K)/ the protein kinase B (also known as Akt) pathway, and the phospholipase C-γ pathway (Figure 1). Binding of neurotrophic factor causes dimerization and autophosphorylation of the Trk receptor leading to activation of signaling cascades through Src and Shc adaptor proteins which are recruited to the Trk receptor. Once activated Shc increases the activation of Ras, which leads to MAPK/ERK1/2, causing differentiation and survival through transcriptional events. Activation of this particular pathway is thought to occur as well in a specialized early endosome, the signaling endosome, which has been shown at least for TrkA to contain various signaling molecules such as PLC-γ, pERK1/2, and the early endosomal protein, Rab5 [5,6]. Transport of the signaling endosome from the axon to the cell body is an important means for transmitting trophic signals to the neuronal soma [7,8]. For sustained MAPK activation, Trk activates the scaffolding proteins Gab2/Shp2 and involves the small G protein, Rap1 on the endosome as well [9]. Shc activation also leads to increases in Akt through activation of PI3K promoting cell survival by inhibiting apoptotic signaling, even though this activation is thought to occur more at the cell surface rather than on intracellular endosomes [10]. These different signaling pathways that are activated by neurotrophins work together to support normal neuronal function and to prevent neuronal cellular death.

## Neurotrophins and neurodegenerative diseases

Given the critical role played by neurotrophins in regulating neuronal functions, it is not surprising then that a significant number of psychiatric and neurodegenerative disorders is associated with altered NGF and BDNF levels and with changed expression of their receptors. For example, neurodegenerative phenotypes similar to Alzheimer’s disease (AD) are observed in a mouse model in which half of the NGF level is neutralized by antibodies [11]. In fact, the brains of AD patients and aged rats show reduced NGF levels in BFCNs [12-14]. Another neurodegenerative disorder, Down’s syndrome (DS), exhibits similar NGF signaling deficits in the same region of the brain [15]. Neurotrophins undoubtly have a strong role in preventing cellular death of BFCNs.

The key role for NGF was discovered in early studies on transected fimbria in which administering NGF upon transection was able to markedly reduce cholinergic neuron death, which was typically induced by the procedure [16]. In addition, NGF administration was found to partially reduce cholinergic atrophy in aged rodents [17]. However, to complicate these neurodegenerative disorders further, alterations in BDNF and its receptor are seen in two very important areas that control spatial memory and higher cognitive function: the frontal cortex (FC) and the entorhinal cortex (EC). Alterations in BDNF in these neurons and the overall selective vulnerability of specific areas to degeneration are seen not only in AD, DS, and normal aging, but also other disorders of the brain pointing to multiple roles for BDNF in particular. Both protein and mRNA levels of BDNF are decreased in dopaminergic neurons of the substantia nigra [18], the neurons most vulnerable in Parkinson’s disease. BDNF has been shown to have survival role here and alterations in BDNF most likely contribute to the disease [19]. In a similar manner, in Huntington’s disease, BDNF transport from cortical to striatal neurons is deficient, contributing to selective loss of striatal neurons and voluntary muscle movements in patients with the disease [20,21]. Moreover, BDNF levels are thought to play an important role in susceptibility of non-neurodegenerative diseases. For various psychiatric disorders like bipolar, depression, anxiety, and schizophrenia, it has been shown that there are abnormal increases and decreases in levels of BDNF throughout the brain and in plasma [22-24]. Strong evidence for the link between these disorders to BDNF specifically originates from data from patients carrying a BDNF variant gene, which contains a methionine mutation in the prodomain. Evidence from these subjects and mouse models carrying the mutation shows smaller hippocampal volumes, along with decreased activity-dependent BDNF release [22].

While it is still unclear as to why certain areas of the brain are more vulnerable in various disorders over other areas of the brain, one effect that is certain is that the synaptic loss and neuronal dysfunction in these areas lead to detrimental changes to overall synaptic transmission [25-27]. Indeed, vulnerable areas in the brain which are impacted the most in many of the diseases mentioned above show a decrease in neuronal size and number, along with reduced expression of neurotransmitter molecules and receptors in response to the decreased trophic support [28]. It is conceivable that cellular and intracellular changes to neurons, induced by alterations in signaling cascades, can impair neuron’s ability to function properly. Alterations in any component along the signaling/survival pathways could potentially exacerbate the deficit in trophic support for neurons, resulting in their dysfunction either locally and/or on a circuit level.

The well documented role for neurotrophic factors to prevent cell death and to maintain cellular function has led scientists to investigate their translational benefit(s). To date, the potential beneficial effect of neurotrophins, NGF and BDNF, in particular, have been explored in light of several neurodegenerative disorders, including but not limited to AD, Amyotrophic lateral sclerosis (ALS) [29], Huntington disease [30], Parkinson’s Disease and even obesity [31] (Table 1).

**Table 1:** Neurotrophic factors that are currently under study for treatment of various disorders

	**Neurotrophic factor**	**Target neurons**	**Current status**
**ALS**	NGF and BDNF	Motor neurons	Recruiting for Phase 1 and Phase 2
**Parkinson’s disease**	GDNF/neurturin	Striatal neurons	Some Phase 1 complete, ongoing in Phase 1 and Phase 2
**Huntington’s disease**	BDNF	Striatal neurons	Pre-clinical
**Alzheimer’s disease**	NGF and BDNF	Cholinergic neurons, entorhinal neurons	Ongoing in Phase 1
**Down Syndrome**	NGF	Cholinergic neurons	Pre-clinical
**Spinal Cord Injury**	BDNF and NT-3	Site of injury	Pre-clinical
**Obesity**	BDNF	Hypothalamus	Pre-clinical
**Lysosomal storage disorders**	BDNF	Various in CNS	Pre-clinical
**Sensory neuropathies**	NGF	Sensory and sympathetic neurons	Phase 2 completed
**Supranuclear Palsy**	GDNF	Various in CNS	Phase 2 completed

Current status defines Phase trials that have either been completed or are underway [32]. Pre-clinical status was assigned to each if the neurotrophic factor has been shown to rescue neuronal functioning in target neurons in rodent and primate models of disease.

## Challenges in neurotrophin-based therapy

Although there are strong rationales suggesting that increasing supply of neurotrophins to degenerating neurons may be a potent way to restore neuronal function in neurodegenerative conditions, delivering neurotrophins into the brain has proven to be a non-trivial matter. Notoriously, CNS diseases are difficult to treat due to the presence of the blood brain barrier (BBB) that makes it almost impossible for large proteins and complex compounds to cross from the blood into the brain. In addition, the cortical and subcortical circuits of the brain are interconnected resulting in crosstalk among multiple regions, so coming up with a treatment strategy that selectively targets affected neurons only, but not those unaffected ones, is a great challenge that has to be carefully considered. To further compound these issues, neurotrophins are relatively large, polar molecules that cannot readily cross BBB and therefore must be administered directly into the central nervous system (CNS). Indeed, all current delivery strategies involve invasive procedures as discussed below.

### Infusion of neurotrophins by direct intracerebroventricular (ICV) injection

To bypass the inability of neurotrophins to cross BBB, purified neurotrophins can be directly infused to the brain by intracerebroventricular (ICV) injection. This delivery route is particularly suitable for NGF to treat BFCN degeneration, since BFCNs extend their axons throughout the hippocampus and neocortex. NGF that is infused into the lateral ventricle can act on the TrkA receptor located at the axonal termini to retrogradely transmit trophic support signal for BFCNs. This approach has been proven especially effective in preventing loss of BFCNs in rodents associated with lesions and aging as mentioned above. However, clinical trial with NGF infusion showed that, although long-term NGF administration by ICV injection may cause certain potentially beneficial effects, the intraventricular route of administration is also associated with significant side effects [33], such as hyperinnervation of cerebral blood vessels [34], hypophagia [33,35], Schwann cell hyperplasia with sprouting of sensory and sympathetic neurons [36], and neuropathic pain [33]. As such, these side effects may cause serious concerns in limiting the dose of infused NGF, thus providing only little therapeutic benefit.

NGF involvement in pain is stemmed from its ability to activate the nociceptive response in sensory neurons [37]. NGF as a therapeutic tool has been particularly impacted by this characteristic, even in attempt to treat peripheral neuropathies such as diabetic and HIV-induced neuropathy, two disorders that do not have the delivery barriers to overcome like those of the CNS. Clinical trials with NGF treatment of these two types of neuropathies have to be terminated due to the fact that severe pains were induced in patients, even though symptoms associated with both disorders were ameliorated in early Phase II studies [38,39]. Even healthy volunteers administered with NGF will begin to feel hyperalgesia at the injection site after 3 hours, with worsening effects over the course of three days [40].

Current efforts with infusion of other neurotrophins have yielded similarly disappointing results. For example, to increase the delivery of BDNF, one study has used intrathecal infusion of N-terminal pegylated BDNF after spinal cord injury. While the authors were able to show that pegylated BDNF was able to reach the spinal cord and induce expression in that area, they saw no improved axonal response or locomotor recovery, suggesting the amount of BDNF that was delivered was still insufficient [41]. In a separate study, although enhanced delivery of BDNF to the CNS was achieved intravenously using a combination of pegylation and conjugation to antibodies targeting the transferrin receptor of BBB, *in vivo* data from this dual approach is still lacking [42]. Intraputamenal infusion of glia-derived neurotrophic factor (GDNF) in Rhesus monkeys has also led to reduced food consumption and weight loss, meningeal thickening and Purkinje cell loss in the cerebellum [43]. More importantly, GDNF infusion provided no significant benefit to human patients with Parkinson’s disease [44]. This reoccurring theme of side effects without significant benefit of treatment has also been shown with intrathecal infusion of recombinant BDNF in patients with ALS [29,45].

### Neurotrophin-producing cell transplantation

**Figure 2:**
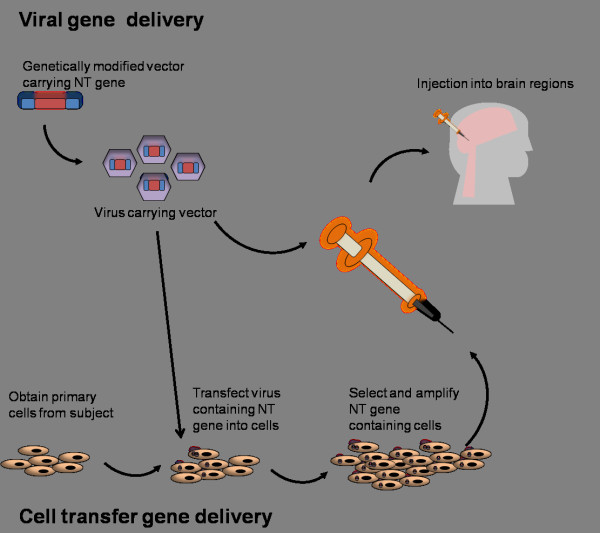
**Gene delivery techniques currently being used to deliver neurotrophin to various sites in the nervous system.** Direct viral gene delivery of neurotrophin (NT) gene occurs through insertion of the neurotrophin into a viral vector and then placement of the viral vector into a host virus, such as adeno-associated virus or lentivirus. Virus is then directly injected into the brain area(s) through surgical techniques. Cell transfer gene delivery first involves obtaining host cells, preferably from the subject and then transfecting them with virus containing the neurotrophin gene. Once selection and amplification of genetically modified cells is performed, and production of the neurotrophin is confirmed, those cells are then injected or grafted into the brain area(s) through similar surgical techniques.

To circumvent the lack of significant therapeutic benefits in combination with serious adverse effects associated with the infusion approach, other methods are designed to achieve more targeted delivery of neurotrophins directly to those populations of neurons affected in disease. This would allow for more controlled and increased dosing while potentially eliminating side effects through avoiding unknown interactions of the neurotrophin. Currently, two approaches of direct delivery of neurotrophic factor into subcortical sites of the brain have been developed and practiced: transfer of cells modified to express neurotrophic factor and delivery of an engineered viral vector encoding the neurotrophic factor protein. The first technique involves establishing cell lines, preferably from the donor host, to express the neurotrophic factor through transfection and selection using vectors containing the gene of interest. Once expression of the gene is assessed and optimized, the cells can be transferred into brain regions to function locally in providing neurotrophic factor (Figure 2). Early proof-of-principle studies for this approach were carried out in 1987 by Gage and colleagues who first established donor rat fibroblast cells to express a prototype HPRT vector then grafted these cells into several regions of the rat brain. They found that HPRT enzymatic activity in the grafted cells remained high for at least 7 weeks after transfer [46]. Following this study, genetically modified fibroblasts secreting NGF were implanted into the brains of rats with fimbria lesions. Not only were the transplanted cells able to survive and produce NGF, they were able to prevent cholinergic loss and cause the surviving neurons to sprout axons towards the direction of NGF-secreting fibroblasts, an indication that the neurons were functioning properly [47]. Similar studies in primate and non-human primates also showed that genetically modified cells from various cell lines (baby hamster kidney and primary cells) were able to rescue cholinergic functioning in injured neurons [48-50]. In addition, this type of gene therapy has been used in both rodent and primate aging models to show that age related reductions in neuronal functioning and memory impairment can be ameliorated through delivery of genetically modified cells that express NGF [51-53]. More importantly, implanted cells sustained NGF production for at least 8 months in the primate brain, and furthermore, administration of NGF in this manner did not elicit those adverse side effects that were seen in infusion studies [52,53], indicating that direct gene delivery could offer a large therapeutic benefit in disease. These findings and ensuing preclinical studies have laid the groundwork that led to the first human clinical trial of NGF gene delivery.

At the beginning of 2001, eight subjects both male and female in early stage AD were enrolled. Primary autologous fibroblasts derived from each subject were genetically modified to produce human NGF using retroviral vectors. Once NGF production was established the cells were injected into the basal forebrain of the subjects either unilaterally or bilaterally. Of those that safely received the NGF delivery, the mean Mini-Mental Status Examination (MMSE) scores showed a mean rate-of-decline reduction of 51 % over a period of 22 months, and an even higher reduction during the 6–18 months when NGF production remained robust. In addition, there were cognitive improvements and increased PET scan activity in several areas of the brain. Post-mortem analysis of one subject which died 5 weeks after NGF delivery confirmed that there was robust NGF expression in the cell grafts and that cholinergic axons showed sprouting. Overall this study provided the first clinical evidence that directed neurotrophic factor delivery could provide therapeutic benefit without side effects commonly associated with neurotrophic factor infusion. Only hemorrhages in two subjects were observed during injection that may be due to unwanted movements during the procedure. One patient did not recover and died shortly after surgery. General anesthesia has been since adopted to mitigate the problem [54]. Currently, a Phase II clinical trial with this approach is underway.

This technique has also been applied successfully for grafting of BDNF and NT-3 in the treatment of spinal cord injuries. Although not yet performed in human patients, fibroblasts that were modified to express BDNF or GDNF and NT-3 and were grafted into sites of spinal cord injury induced sustained regenerative and sprouting responses into the sites of injury in rats [55,56]. Overall direct gene delivery in the clinical setting may prove to have the most beneficial impact yet, this type of procedure remains an invasive technique. Furthermore, although grafted cells have been shown to be functional for up to one year after implantation, subsequent injections may have to be given over the course of a subjects lifetime in order to sustain benefits from the treatment. Moreover, the long term effect(s) of the presence of the large number of fibroblasts in the brain needs to be fully evaluated.

### Viral vector-mediated gene delivery

Due to advances in molecular research in the past decade, viral vector-mediated gene delivery may prove to be a more optimal approach (Figure 2). Delivery of a virus would confer a permanent change in the ability of the neuron to make its own neurotrophic factor, leading to a single injection at the site instead of multiple injections, therefore decreasing the invasiveness. Intrastriatal injection of adeno-associated virus (AAV) vector encoding an enzyme essential in the production dopamine, aromatic L-amino acid decarboxylase (AADC), into MPTP-lesioned non-human primates resulted in expression of the enzyme for at least six years [57]. Even more appealing in viral delivery is that the cumbersome cell preparation associated with the cell transfer technique would be eliminated and that AAV vectors do not express their own proteins and therefore would not elicit an immune response. To date, viral delivery has been used and evaluated in a number of rodent, primate and human subject studies, particularly for Parkinson’s disease (PD). A hallmark of PD is specific dopaminergic loss in the striatum, leading to neuronal and motor dysfunction. Viral gene delivery of AAV encoding AADC was shown to provide eight years of clinical improvement in non-human primates, one of the longest *in vivo* studies that have been performed thus far [58]. Similarly, viral delivery of GDNF by lentivirus reversed motor deficits in MPTP-treated monkeys and prevented nigrostriatal degeneration [59]. AAV-mediated delivery of an analog of GDNF, neurturin, has also been shown to be neuroprotective for dopaminergic neurons in rats [60]. Studies have demonstrated that injection of the neurturin viral vectors is safe, tolerable and could potentially lead to improvements in motor functioning of actual Parkinson’s disease patients at 1 year and in rhesus macaques [61,62]. However, as with cell-mediated gene delivery, a small number of human subjects that were given the injection suffered from intracranial hemorrhages [63], indicating that more surgical training and care, perhaps even better injection techniques, need to be adapted to make this type of treatment more applicable.

Viral treatment has also been explored for treating other disorders such as the lysosomal storage disease, late infantile neuronal ceroid lipofuscinosis [64] in which child subjects demonstrated a reduced rate of neurological decline. In addition, administration of a lentiviral construct expressing BDNF into rodent and primate models of AD showed improved cell signaling and a restoration of learning and memory, while reversing synaptic loss [65]. Lentiviral NGF gene delivery in rats has been just as beneficial in preventing cholinergic neuron loss upon fimbria-fornix lesion injury [66]. Currently ongoing and future clinical trials in human subjects using both BDNF and NGF viral delivery should inform about their safety and efficacy, and their potential benefits.

## Neurotrophin-peptide mimetics/ small molecules with neurotrophic properties

It is worth mentioning that many of the challenges facing either direct infusion of neurotrophic factor or cell-, viral vector-mediated gene delivery methods may be overcome with small molecules that target the receptor for the neurotrophic factor instead of introducing the neurotrophic factor itself. This would allow for specific activation of only one type of receptor, such as TrkA or TrkB and not p75, or vice versa, potentially alleviating the side effects. To this end, the discovery and use of peptide mimetics, short peptides that have improved bioavailability and lower degree of proteolysis [67], and small molecules ligands for the Trk receptors [68] have attracted intensive interest. A potent peptide mimetic of BDNF has been shown to activate TrkB, promoting neuronal survival in embryonic chick dorsal root ganglion sensory neurons [69]. Also, small molecule BDNF mimetics have high potency and specificity towards TrkB and can promote neuronal survival as well, while also inducing differentiation and synaptic function in cultured hippocampal neurons [70]. When administered to mouse models of AD, Huntington, and PD, the small molecule was able to rescue cellular death to the same extent of full protein BDNF [70]. Further studies using small molecule mimetics of BDNF confirmed their broad application in both restoring TrkB function and improving respiratory function in mouse models of Rett Syndrome [71] and in facilitating functional recovery after stroke while promoting an increase in the number of neurons adjacent to stroke site [72]. Currently, a number of clinical trials are being carried out using neurotrophic factor mimetics [68]. Results from these trials, especially concerning side effects and efficacy, will broaden and enhance neurotrophic factor -based therapy for treating neurodegenerative disorders.

### Combinational therapy using neurotrophins and other small molecules

Neurodegenerative disorders are very complex diseases. Although neurotrophic factor-based strategies have offered great potential, the biggest unknown is whether such approach by itself is adequate in halting and reversing the course of progression of these diseases.

As years have passed and many clinical trials later, the idea that “a single magic bullet approach” or one drug can act as the sole solution for treating neurodegenerative disorders has proven not very successful. This is highlighted well in the case of Alzheimer’s disease. For example, acetyl cholinesterase inhibitors (Aricept) and N-methyl-D-Aspartate (NMDA) receptor antagonists (Mementine) have been approved for the treatment of AD, but both treat symptoms and show only moderate efficacy. Unfortunately, both fail to slow the rate of cognitive decline in AD patients [73]. Furthermore, another painful lesson came from the recent failure of a Phase III trial using gamma secretase inhibitors to treat AD, a “disease-modifying” compound which has been a sought after drug target for some time [74]. It is clear that various treatments with small molecule drugs such as these, have yielded only modest results at best. Novel small molecules for AD, including disease-modifying gamma secretase modulators, are currently under extensive evaluation for their potential for AD treatment [75]. Given past failure in monotherapy in this arena, it may become necessary to use a combination of approaches, i.e. combinational therapies, to attack the different disease causing mechanisms simultanously. We can envision that by a combinational use of a small molecule with neurotrophins may work synergistically to restore neuronal function and to minimize possible side effects as discussed above.

Going forward, the idea of combinational therapies for treating various neurodegenerative disorders is worth serious consideration, given the fact that so many attempts with monotherapies have not yielded any success. For many non-neurodegenerative disorders co-therapies targeting multiple disease pathways and symptoms are actively being used and evaluated. For rheumatoid arthritis, disease modifying antirhematic drugs (DMARDS) are used in combination with fast acting glucocorticoids to alleviate the symptoms of inflammation quickly [76]. The combination of DMARDS and glucocorticoids was shown to cause a reduction in both the tolerability and side effects of DMARD infusion alone [76-79]. Moreover combinational therapy using various DMARDS and glucocorticoids together resulted in short and long term improvements when compared to DMARDS alone [76]. For cardiovascular disease involving cholesterol, treatment with statins is the standard. However, statins have shown over time to eventually lead in some cases to regression of the disease [80]. Co-therapy using statins and niacin have shown to lead to significant decreases in disease causing low-density lipids, while raising beneficial high-density lipids [80,81] which may lead to tighter lipid control and therapeutic benefit for those with statin-treated cardiovascular disease. Even for diabetes, insulin, which has been the main glucose lowering treatment, has been evaluated in combination with recombinant human insulin-like growth factor I (IGF1), a pathway that if restored can lead to much higher glucose lowering than insulin alone. Subjects receiving both IGF1 and insulin together decreased their insulin need, while those treated with insulin alone had an increase in insulin usage [82]. Examples such as these highlight various properties of combinational therapies in treating a disorder and confirm why co-therapy in neurodegenerative disease may prove to be most successful. First, these examples show that one can quickly relieve symptoms of a disease with one or more drugs while concomitantly treating the disease itself with another. This is important since treating a disease will most likely have a longer time course inherent to its action than solely relieving symptoms. Secondly, combinational therapy using two or more drugs can compensate for one drug’s inactivity over time and therefore potentially inhibit regression of the disease. The final property illustrated here is that combinational therapy using two or more proteins/small molecules can work synergistically together so that one or both are needed in lower dose or less frequently, a convenience that someone suffering from an illness would definitely benefit from.

## Conclusions

NGF and its other family members provide potent trophic support to neurons. Their robust effects in rescuing degenerating neurons cannot be matched in this regard by any small molecules or compounds identified thus far. Neurotrophin-based therapies may well prove to be an effective means to combat epidemic neurodegenerative diseases. Yet, many daunting challenges remain to be resolved. Furthermore, it remains to be seen if such strategies that aim at a single target will be sufficient to cure these diseases. In the end, the inherent complexity of neurodegenerative diseases may require combinational therapies that target multiple pathways for effective treatment.

## Competing interests

The authors declare that they have no competing interests.

## Authors’ contributions

AW drafted the manuscript, CW critically revised the manuscript. Both authors read and approved the final manuscript.
